# Comparing Manufacturer Submitted and Pan-Canadian Oncology Drug Review Reanalysed Incremental Cost-Effectiveness Ratios for Novel Oncology Drugs

**DOI:** 10.3390/curroncol28010060

**Published:** 2021-01-20

**Authors:** Ronak Saluja, Tina Jiao, Liza Koshy, Matthew Cheung, Kelvin K. W. Chan

**Affiliations:** 1 Odette Cancer Centre, Sunnybrook Health Sciences Centre, Toronto, ON M4N 3M5, Canada; ronak.saluja@mail.utoronto.ca (R.S.); tina.jiao@uwaterloo.ca (T.J.); lkoshy@uwaterloo.ca (L.K.); Matthew.Cheung@sunnybrook.ca (M.C.); 2 Department of Medicine, University of Toronto, Toronto, ON M5S 1A8, Canada; 3 Canadian Centre for Applied Research in Cancer Control, Toronto, ON M5G 2L3, Canada; 4 Cancer Care Ontario, Toronto, ON M5G 2L7, Canada

**Keywords:** cost-effectiveness models, pan-Canadian Oncology Drug Review (pCODR), incremental cost effectiveness ratio (ICER), time horizon, drug wastage

## Abstract

Background: To determine the magnitude of difference between manufacturer-submitted and pan-Canadian Oncology Drug Review (pCODR) calculated incremental cost-effectiveness ratios (ICERs), incremental cost (ΔC), and incremental effectiveness (ΔE); to examine whether there is a significant difference in the proportion of ICERs deemed cost-effective; to evaluate trends in the ICERs over time; and to identify methodological issues in manufacturer-submitted economic models. Methods: Economic guidance reports for all drug indications submitted from July 2011–November 2018 were extracted from the pCODR database. Cumulative distribution plots were constructed to compare the manufacturer-submitted economic values with both the pCODR lower- and upper-reanalyzed estimates. The proportion of drug reviews considered cost-effective at varying willingness-to-pay (WTP) thresholds by the manufacturer and pCODR were calculated. Manufacturer changes in ICERs over time from 2012 to 2018 were determined. Recurring methodological issues with manufacturer submissions were tallied. Results: There were 73 unique indications that were included. Manufacturer-submitted ICERs were consistently lower than pCODR estimates for most indications. Manufacturer-submitted ICERs were generally more cost-effective over a range of WTP thresholds. From 2012 to 2018, manufacturer and economic guidance panel (EGP) lower limit reanalyzed ICERs did not change significantly over time. However, EGP upper limit re-analyses did show decreasing cost-effectiveness (increasing ICERs). The two most common issues identified in the manufacturer-submitted models were related to survival time horizon and utility estimates. Conclusions: Manufacturers tend to overestimate the cost-effectiveness of their therapies when submitting economic models to pCODR. Although certain methodological issues are still common in manufacturer-submitted models, revision rates are high for most issues raised by pCODR.

## 1. Introduction

Anti-cancer therapies are a significant financial burden on both patients and healthcare systems. As the incidence of cancer continues to rise and new therapies are rapidly introduced, public drug plans with limited budgets continue to face difficult resource allocation decisions [1]. Assessing cancer drugs for public reimbursement recommendation is a complex evidence-based process. In Canada, the pan-Canadian Oncology Drug Review (pCODR), originally established in 2011 and now a part of the Canadian Agency of Drugs and Technologies in Health (CADTH), is a pan-Canadian cancer specific health technology assessment (HTA) process created to make funding recommendations to federal, territorial, and provincial drug plans (except for the province of Quebec) [2]. The pCODR Expert Review Committee (pERC) reviews submissions from drug manufacturers and makes non-binding recommendations, integrating the overall clinical benefit, patient perspectives, cost-effectiveness, and feasibility of adoption of the drug into the health system into their decision [3].

Because of limited healthcare resources, evidence of cost-effectiveness is a critical component of drug funding decisions [4]. Novel therapies are considered “dominant” and are preferred if they are less costly and more effective than the alternative [5]. However, in almost all cases, new treatments that are more effective than existing therapies are often costlier. When evaluating these “non-dominant” drugs, the calculated incremental cost-effectiveness ratios (ICERs) can be compared to a willingness-to-pay (WTP) threshold, commonly cited as being between $20,000–$100,000 CAD per quality-adjusted life year (QALY) in Canada, albeit potentially controversial and arbitrary [5,6,7]. A drug is considered cost-effective if its calculated ICER falls below the WTP threshold [6]. Unlike some other countries and HTA bodies, it should be noted that Canada and pERC have not adopted an explicit WTP threshold.

In the pCODR review process, the cost-effectiveness data used in pERC’s deliberations are delineated through a two-step submission process, as follows: manufacturers first submit their economic models to pCODR, specifying their best estimate of the drug’s ICER, ΔC, and ΔE. pCODR subsequently convenes an economic guidance panel (EGP), consisting of independent health economists or HTA-experienced panel members [3,8]. The EGP reanalyze the manufacturer’s model, revising components contributing to potential uncertainties and limitations. The revision process allows the EGP to calculate their best upper and lower estimates of ICERs, incremental cost (ΔC), and incremental effectiveness (ΔE). Manufacturers are permitted to review and provide feedback on the economic guidance reports prior to pERC making its final funding recommendation [3].

Previous studies have examined the methodological issues in manufacturer submitted economic models to pCODR, the components commonly revised by the EGP, and their potential correlation with final pERC recommendations [9,10,11]. Masucci et al. described the most frequent methodological issues identified and revised by EGPs, which involved costing (59%), time horizon (56%), and model structure (36%) [11]. None of these issues, however, had a statistically significant relationship with the pERC’s final recommendations [11]. The final recommendations are more dependent on clinical evidence and patient values, while subsequent pricing negotiations may be more dependent on cost-effectiveness [7]. Two other studies evaluating methodological issues in manufacturer-submitted economic models found that the most common revision by the EGP was the shortening of the time horizon [9,10].

Identifying the recurring problems faced by economic models submitted to HTA review committees is an important step in the development of more suitable models in the future. While previous studies have examined some of these methodological issues, to our knowledge, a direct comparison of manufacturer-calculated and independently reanalyzed ICERs has not been formally conducted in the Canadian context. Therefore, in this study, we investigate if manufacturer-submitted economic models are generating ICERs that are more optimistic in comparison with pCODR-generated ICERs. The objectives of this study are to determine the magnitude of difference between manufacturer and pCODR ICERs, ΔC, and ΔE; to examine whether there is a significant difference in the proportion of ICERs deemed to be cost-effective; and to evaluate the trends in the ICERs over time. To further explore why manufacturer-submitted and EGP-reanalyzed ICERs may differ, we also present more recent data on the common methodological issues revised by the EGP.

## 2. Methods

### 2.1. Selection of Drug Indications

All drug indications submitted from 13 July 2011, when pCODR first started accepting submissions, to 1 November 2018, were identified from the publicly available pCODR database [12]. Indications with a final economic guidance report that stated both the manufacturer ICER/ΔC/ΔE and the EGP’s lower and upper estimates for ICER/ΔC/ΔE were included. For duplicate entries due to a resubmission, the most recent submission was included. Indications with redacted or unreported ICERs, ΔC, or ΔE for either the manufacturer or EGP reanalysis were excluded. Submitted indications that did not report point estimates but only a range in the manufacturer-submitted ICER, ΔC, or ΔE were excluded. Indications where both the manufacturer and the EGP considered a drug as “dominant”, regardless of if ΔC or ΔE were reported, were included. If only one economic model deemed the drug as “dominant”, the other must have provided an ICER to be included in this analysis. In cases where there were multiple indications in a single review or more than one ICER for a single indication due to multiple comparators, the most appropriate comparator (as per the pCODR final clinical guidance reports, which reflect the most relevant Canadian comparator) was extracted, as pCODR only provides upper and lower estimates for this most relevant comparator.

### 2.2. Data Extraction

For each drug review, two reviewers (T.J. and R.S.) independently extracted the generic drug name, pCODR number, indication, route of administration, comparator drug, pERC final recommendation (positive, positive with conditions, or negative), date of final recommendation, manufacturer-submitted economic values reported as point estimates (ICER, ΔC, and ΔE), and EGP’s lower and upper estimates (ICER, ΔC, and ΔE). Methodological issues were identified by examining final economic guidance reports, and recurring issues were grouped into categories based on theme and whether the issue was revised by EGP during reanalysis. To ensure accuracy, all data extractions were compared between the two reviewers, with disagreements being resolved through discussion with a third reviewer (K.C.).

### 2.3. Statistical Analyses

Key data from included drug reviews, including the ICER, ΔC, and ΔE values, were summarized using descriptive statistics. Cumulative distribution plots were constructed to compare the manufacturer-submitted economic values with both the lower and upper EGP reanalyzed estimates. The absolute differences in ICERs were calculated by subtracting the manufacturer-submitted value from the EGP reanalyzed estimates; therefore, a positive difference indicated that the manufacturer’s ICERs were lower and more optimistic. The proportion of drug reviews considered to be cost-effective at varying willingness-to-pay (WTP) thresholds (from 50,000 to 150,000 $/QALY) by the manufacturer and EGP reanalyzed models were also calculated. As Canada does not endorse an explicit societal WTP threshold, a range was used in this analysis based on the World Health Organization’s suggestion of one to three times gross domestic product per capita [13]. ICERs that fell below the WTP threshold were considered cost-effective. Generalized linear regression models with Gamma distribution and log link were used to examine the changes in ICERs over time from 2012 to 2018, as costs have non-negative values and are right-skewed [14,15]. Lastly, methodological issues with manufacturer submissions that lead to discrepancies between manufacturer-submitted and EGP reanalyzed estimates were extracted from the economic guidance reports and were tallied. The percentage of these issues that were revised by the EGP prior to making their recommendations were also calculated. All statistical analyses were performed using R (version 3.2.0, R Foundation for Statistical Computing, Vienna, Austria).

## 3. Results

**Figure 1:**
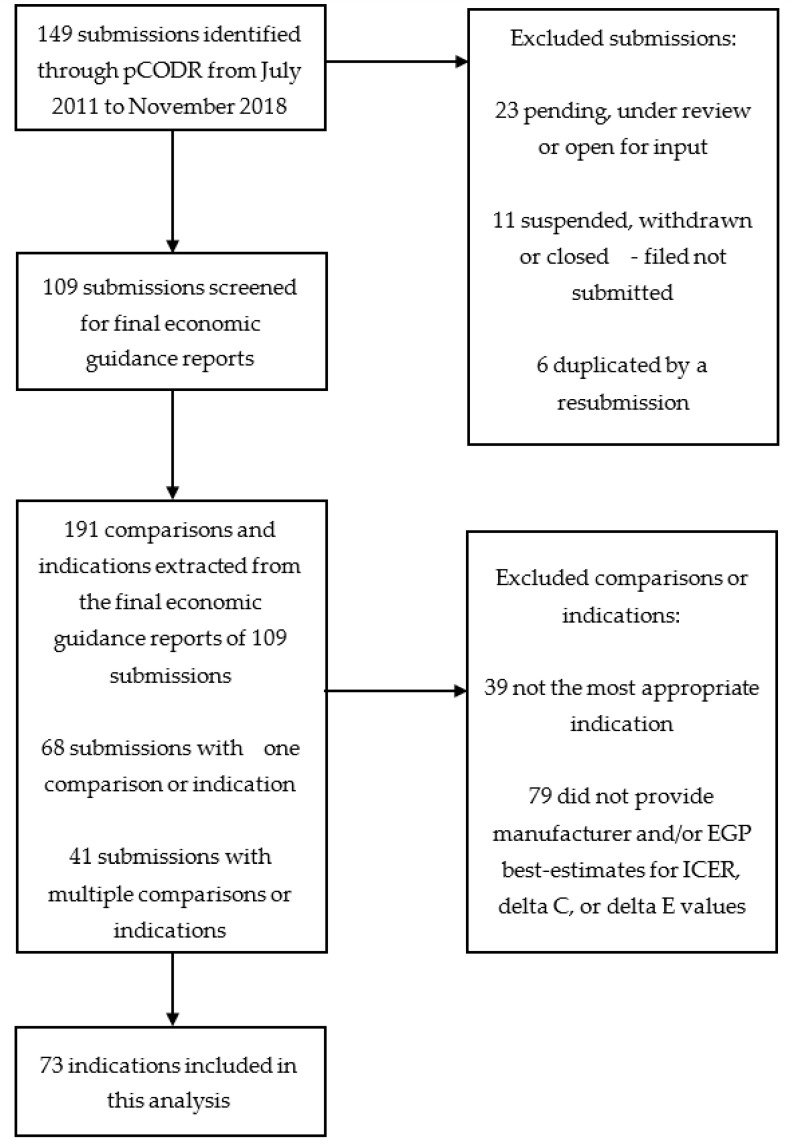
Selection of indications. For certain submissions, multiple indications were reported and only the most appropriate indications as per the pan-Canadian Oncology Drug Review (pCODR) Final Clinical Guidance Reports were included in these cases.

Overall, 73 unique indications were included in this analysis, from 149 submissions initially identified from pCODR (Figure 1). No indications from 2011 were eligible for inclusion. The included indications covered a variety of cancer types, with the most common being leukemia (16%), lung cancer (14%), and gastrointestinal (14%) cancer. The characteristics of the included indications are summarized in Table 1 and Supplementary Tables S1 and S2.

### 3.1. Differences in Manufacturer-Reported and EGP-Reanalyzed Values

**Figure 2:**
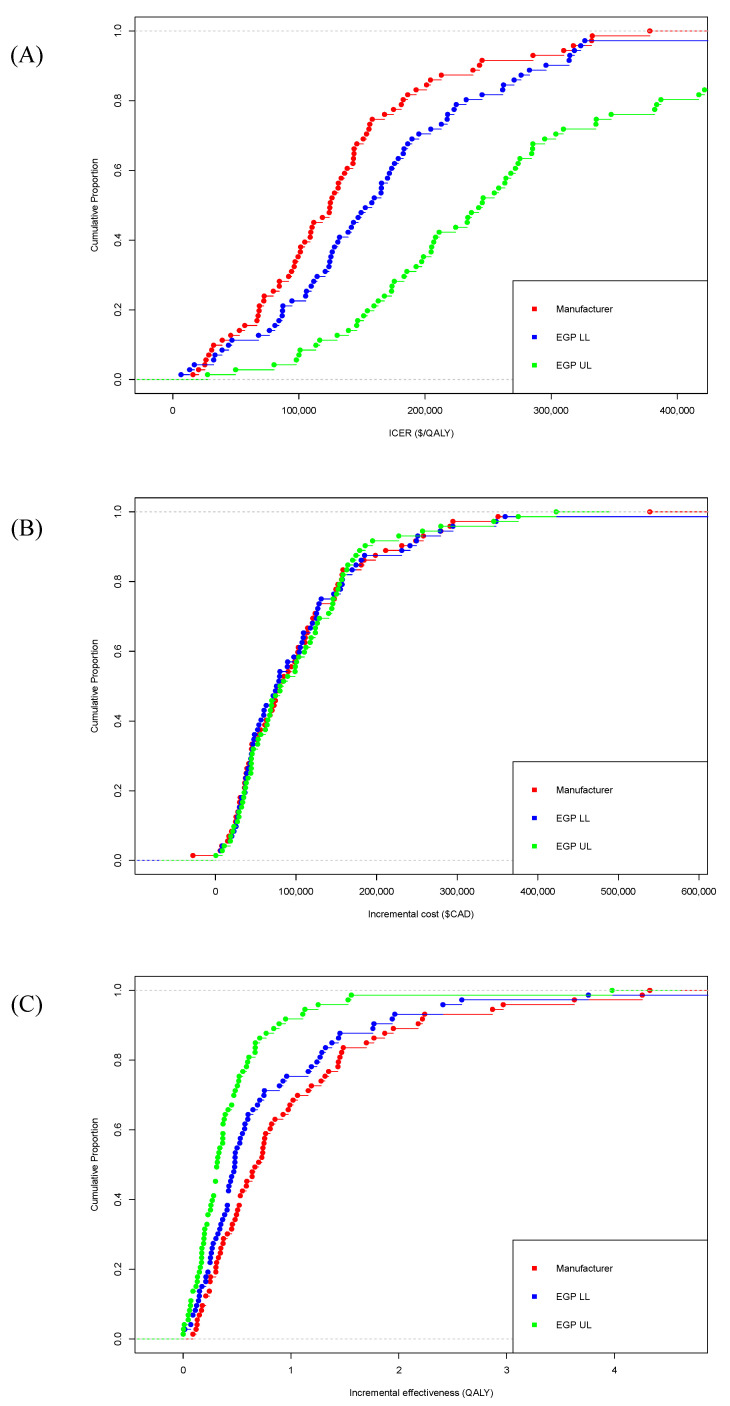
Cumulative distribution plots of manufacturer-submitted and economic guidance panel (EGP)-re-analyzed (**A**) incremental cost-effectiveness ratios (ICERs), (**B**) incremental cost, and (**C**) incremental effectiveness for each indication. EGP—economic guidance panel; LL—lower limit; UL—upper limit; ICER—incremental cost-effectiveness ratio; QALY—quality-adjusted life year.

**Figure 3:**
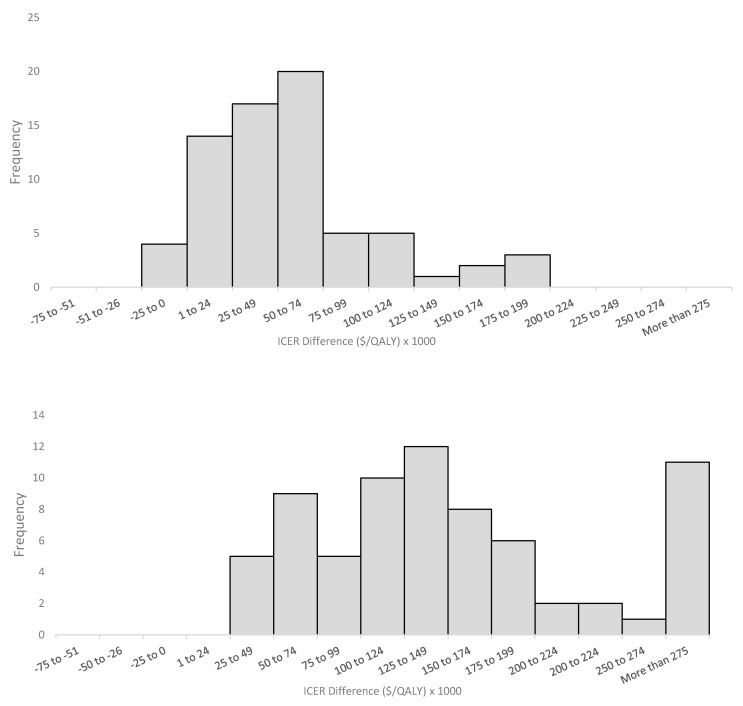
Histogram of differences between EGP-reanalyzed and manufacturer-submitted ICERs. (**Top**) EGP lower limit - manufacturer-submitted ICERs; (**bottom**) EGP upper limit - manufacturer submitted ICERs. Positive differences indicate higher EGP re-analyzed ICERs. Excluded ICERs for all three economic models if one economic model deemed the drug as dominant (1) or dominated (1) and did not provide a numerical ICER value. When comparing the EGP upper limit and manufacturer submitted ICERS (B), four indications had differences greater than $500,000/QALY. EGP—economic guidance panel; ICER—incremental cost-effectiveness ratio.

In examining the ICERs, manufacturer-submitted ICERs were consistently lower than both the EGP low and high estimates for most indications (Figure 2A). The mean ICERs for manufacturer-submitted, EGP low estimate, and EGP high estimate were $134,241/QALY, $166,382/QALY, and $365,018/QALY, respectively (Supplementary Table S3 and Supplementary Figure S1). The distribution of differences between both EGP-calculated ICERs and manufacturer-submitted ICERs are presented in Figure 3. In total, 19 (26%) manufacturer-submitted ICERs fell within the EGP reanalyzed range. There was no difference in EGP low ICER estimates and manufacturer-submitted ICERs in four cases (see Supplementary Tables S1 and S2, pCODR #s 10028, 10008, 10025, and 10055). Manufacturers reported greater (negative difference) and lower (positive difference) ICERs in 14 and 52 cases, respectively. Compared with EGP high ICER estimates, the manufacturer-reported ICERs were lower (positive difference) than the EGP values in all of the cases (Supplementary Figure S1).

Separating the ICERs into their two components (ΔC and ΔE), it was observed that the values for ΔC were generally similar between all three economic analyses (see Figure 2B). In terms of effectiveness, manufacturer-submitted ΔE values were consistently higher than both the EGP low and high estimates (see Figure 2C). Overall, EGP re-analyses generally agreed with the manufacturer with respect to the incremental cost, but disagreed with respect to incremental effectiveness, resulting in substantial disagreements in ICERs.

### 3.2. Cost-Effectiveness Status of Manufacturer Generated and EGP Reanalyzed ICERs

**Figure 4:**
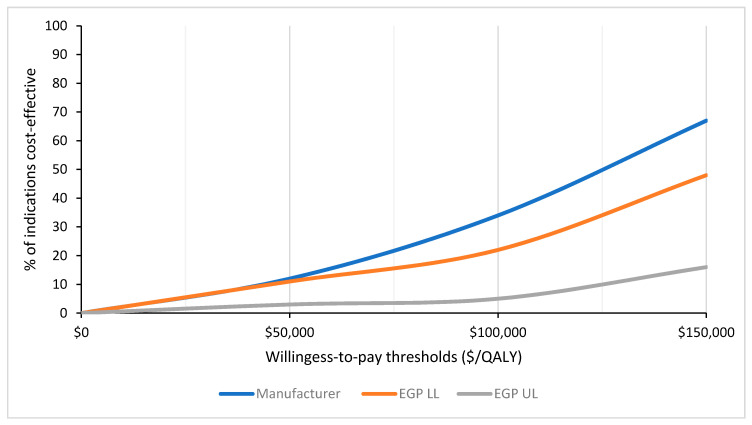
Percentage of cost-effectiveness indications, at varying willingness-to-pay thresholds, based on incremental cost-effectiveness ratios generated from the manufacturer-submitted and EGP-re-analyzed economic models. EGP—economic guidance panel; LL—lower limit; UL—upper limit; ICE—incremental cost-effectiveness ratio; QALY—quality-adjusted life year.

Using manufacturer-submitted ICERs, more interventions were considered cost-effective over a range of willingness-to-pay thresholds (Figure 4). Based on a threshold of $100,000/QALY, 25 indications (34%) were deemed cost-effective by manufacturer-submitted ICERs, 16 (22%) using the EGP lower-limit ICER estimates, and only 4 (5%) with the EGP upper-limit ICER estimates. In the manufacturer submitted data, one indication was considered dominant (pCODR # 10030), while both the EGP lower-limit and upper-limit re-analyses considered one indication dominated (pCODR #10061) by the standard treatment (see Supplementary Tables S1 and S2). At WTP thresholds of $50,000 and $150,000/QALY, 9 (12%) and 49 (67%) indications were considered cost-effective by the manufacturer, respectively. Comparatively, EGP lower and upper limit re-analyses considered 8 (11%)/35 (48%) and 2 (3%)/12 (16%) of indications cost-effective at these two WTP thresholds, respectively.

### 3.3. Change in Manufacturer-Submitted and EGP-Reanalyzed ICER Over Time

From 2012 to 2018, a trend towards decreasing manufacturer-reported ICERs over time was observed, but this was not statistically significant (*p* = 0.78; Supplementary Figure S2). In the EGP re-analyses, EGP low estimate ICERs did not significantly increase over time (*p* = 0.94), while ICERs in the EGP high re-analyses increased significantly by 19% annually (*p* < 0.05; Supplementary Figure S2). Both the manufacturer-submitted and EGP low estimate data suggest a maintenance of ICERs over time. However, EGP high estimate data show a potential worsening of cost-effectiveness with increasing ICERs over time.

### 3.4. Issues with Manufacturer-Submitted Economic Models

**Figure 5:**
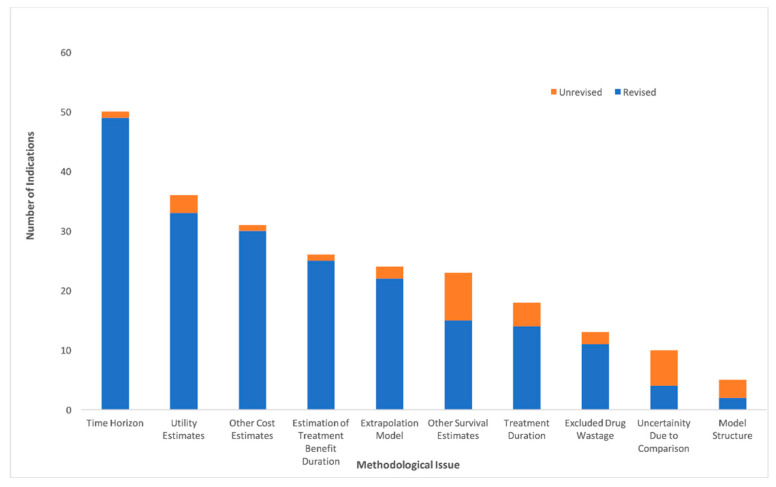
Common methodological issues in manufacturer-submitted economic models identified and revised by the economic guidance panel.

Issues identified with the manufacturer-submitted models were grouped into 10 thematic categories (Figure 5). The two most common issues identified in these models were related to time horizon (50 indications), where the manufacturer chose a time frame that was considered too long for the estimate of the maximum survival of patients, and utility estimates (36 indications) where alternative sources, values, or assumptions were made in the re-analysis. Time horizon was also the most common methodological issue revised by the EGP (lifetime time horizon for 49 of the 50 indications was shortened). This was followed by “other cost estimates” that includes all costs (i.e., treatment, testing, and administrative costs) not pertaining to drug wastage, which refers to the wastage of medications, for instance from discarding leftover intravenous drugs in partially-used vials. Two issues that were identified by EGP but not commonly revised/re-analyzed pertained to “uncertainty with comparisons” and “model structure”. Uncertainty due to an indirect comparison, for instance lack of homogeneity or consistency in the trials used for indirect comparison, was present in 10 indications, with the EGP revising four indications by using a different comparator. Model structure issues, such as bias while using partitioned survival models that require significant extrapolation, were identified in five, but only revised in two indications. Other less common issues included estimation of the treatment benefit duration, which refers to the length of time the drug is expected to provide benefit, including in some cases a post-progression benefit, which was not clinically supported. Extrapolation issues referred to concerns with the statistical techniques used for extrapolation, such as improper survival curve fitting using inappropriate distributions for the clinical data.

## 4. Discussion

Manufacturer-submitted ICERs were consistently lower than EGP low and high limit estimates, suggesting that manufacturers may be overestimating the cost-effectiveness of their drugs. While these findings could also be potentially interpreted as EGP under-estimating the cost-effectiveness of those drugs, given that the EGP for each submission is comprised of academic researchers independent of manufacturers and payers, and different researchers are involved across the different submissions, it is unlikely that the pCODR process would produce biased estimates of ICERs that consistently underestimate cost-effectiveness. Furthermore, concerns about the accuracy of manufacturer-submitted economic models have been ongoing, and were also raised previously by the Joint Oncology Drug Review (JODR) review committee, the precursor to pCODR. A previous study that examined issues encountered by JODR when evaluating manufacturer-submitted economic models reported an uncertainty of comparative clinical benefits, costing assumptions that favored manufacturers and a lack of robustness for the submitted analyses to be the most common concerns [16]. Manufacturer-submitted models also considered a higher proportion of cancer drugs to be cost-effective based on a variety of WTP thresholds when compared to the EGP reanalyzed estimates. This incongruity between manufacturer-submitted models and the EGP reanalysis was also highlighted by Raymakers et al., who found only 11 of the included 43 (26%) pCODR submissions had manufacturer-submitted ICERs that fell within the EGP calculated range [17]. While this study included a limited sample size, our analysis of 73 submissions further confirms this discrepancy between the manufacturer-submitted and EGP reanalyzed ICERs. Similar to Raymaker et al., only 19 (26%) manufacturer-submitted ICERs in our study fell within the EGP reanalyzed range. However, in contrast to Raymakers et al., who cited drug prices as the primary driver of the manufacturer ICERs, we found incremental gains in QALYs have a larger influence. While incremental cost values were similar in manufacturer-submitted and EGP re-analyzed models, manufacturers tend to incorporate higher incremental effectiveness. Thus, the improved cost-effectiveness reported by manufacturers is likely due to an overestimation or optimistic extrapolation of clinical effectiveness, rather than an underestimation of cost. These findings are similar to Cressman et al., who found only 25% of manufacturer-submitted models had higher rates of ΔC, while 72% reported higher rates of ΔE compared with the National Institute for Health and Clinical Excellence’s (NICE) Assessment Group estimates in the U.K. [18].

Over the six-year period analyzed, the trend in cost-effectiveness was similar between the manufacturer and EGP lower limit re-analyses, with ICERs not significantly changing over time. However, the EGP upper limit re-analyses did show decreasing cost-effectiveness (increasing ICERs) over this six-year period. Previous research has shown that the cost-effectiveness of novel anticancer drugs is decreasing over time, as the cost of these therapies has been increasing dramatically without a proportional increase in clinical effectiveness [18,19]. These trends are congruent with EGP’s upper limit re-analyses, but not with the cost-effectiveness trends seen in the EGP lower limit and manufacturer submitted data.

In terms of the methodological issues with manufacturer-submitted economic models, time horizon issues were the most commonly identified and revised by the EGP. The EGP commonly re-computed the ICERs using a different (lifetime) time horizon, especially in cancer settings, where the models inappropriately projected clinically implausible survival tails of 30 to 40 years. These “long and thick” extrapolated survival tails are not properly validated by data, but are unfortunately created by the submitting modeler under the misguided defense of a “lifetime horizon”. In cases where submitted models project a substantial proportion of patients surviving many years beyond the expected survival based on data and clinical experience in these cancer settings, EGPs will adjust the model to a different lifetime time horizon in order to minimize the inappropriate effects of the overestimation of long-term survivors on the cost-effectiveness results. Overall, submitted models that incorporate inappropriately long time horizons may overestimate long-term survival benefits. This is similar to Masucci et al., suggesting that manufacturers continue to overestimate survival when submitting their drugs to pCODR [11]. Issues with survival estimation were also highlighted by a study of 45 HTAs undertaken for NICE in the U.K., with findings that survival analysis is not conducted in a systematic way, and inappropriate survival models are frequently used in submitted HTAs [20]. Similarly, studies from both France and Australia evaluating submitted economic evidence highlighted common issues related to clinical efficacy and extrapolating beyond the time horizon [21,22]. Costing and utility estimation issues are also frequently present in economic models submitted by the manufacturer to pCODR. Again, prior research has similarly documented concerns that manufacturers tend to make more optimistic assumptions in their model inputs with respect to costing and utility estimates [11]. Raymakers et al. reported that manufacturers do not consistently collect health-related quality of life data in their clinical trials in order to calculate QALYs, but tend to rely on lower quality evidence from previous studies [17]. Thus, concerns regarding utility estimates or data used to calculate QALYs in the economic models submitted is an ongoing concern. Issues related to model structure were mentioned less commonly in more recent submissions. While time horizon, costing, and utility estimation issues still exist, these model inputs can be more easily revised by reviewers through sensitivity analyses, unlike issues related to the model structure. Recognizing the need for more transparency in order to address the more difficult methodological issues such as the model structure, CADTH continues to publish revised guidelines for the economic evaluation of health technologies that submitters are advised to consult [23]. In contrast to Massuci et al., who grouped drug wastage with other costing issues such as dosing and pricing structure, administration, and test costs, drug wastage was uniquely accounted for as a separate methodological issue in our study, with 13 (18%) manufacturer submissions failing to report any drug wastage data. Drug wastage can have a considerable impact on economic evaluations, with the potential to significantly increase ICERs [24]. Our results are congruent with previous research showing that drug wastage is still not uniformly considered in economic models.

Our study is not without limitations: firstly, our analysis included 109 economic guidance reports with 73 indications due to the exclusion of submissions that did not publish all of their ICER data. While incorporating these additional indications might impact our findings, a potential limitation is that it is unclear whether the excluded reviews are methodologically similar to those included. Nevertheless, there is insufficient publicly available data for these excluded submissions to allow for further in-depth analysis. This highlights the importance of transparency in the reporting of economic models that inform public-funding decisions in order to enhance accountability and public scrutiny. Secondly, the categories used to classify commonly identified issues in our study were chosen subjectively based on how frequently they were reported in the economic guidance reports. Therefore, any issues discussed by the EGP and manufacturers (i.e., by verbal communication) that were not published in these reports were not captured. Another limitation is that EGP provides upper and lower limit estimates of ICERs, without providing a best point estimate in many cases. In certain indications, the difference between EGP upper and lower estimates can vary widely, making the comparison between the EGP and manufacturer ICERs less precise. Furthermore, there is also no evidence to show that EGP re-analyses are necessarily the gold standard for comparison, but the consistent finding of higher EGP ICERs that were derived from multiple health economists and academic researchers independent of manufacturers and payers across most submissions suggested that these ICERs were less likely to be subjected to perceived or actual influence from any stakeholders. For instance, there is still controversy regarding the appropriateness of adjusting the time horizon in the submitted economic models. Ideally, the time horizon should represent a life-time horizon, but two main issues emerge, namely: (1) there is no consensus on the correct life-time horizon for different cancer settings, and (2) most randomized controlled trials have a short-term follow-up, limiting the extent to which clinical benefits can be extrapolated. In revising the economic models, EGP may reduce the time horizons to remove future projected benefits that were unproven and subject to considerable uncertainty. However, there is no clear consensus if this approach taken by the EGP is the ideal approach to explore uncertainty in the over-extrapolation of potential long-term benefits when only short-term data exist. Finally, economic studies for oncology drugs conducted in the province of Quebec were not included in this analysis, as pCODR does not provide drug funding recommendations to Quebec. Future studies can be performed to expand this analysis to include economic reports from Quebec, and to compare our current results. As the number of submissions to pCODR increases and the sample size becomes larger, other areas of future research include evaluating methodological issues in submitted economic models based on specific disease areas or drug types (i.e., evaluating model issues of newer therapeutics such as immunotherapies).

With the increasing cost of oncology drugs, manufacturer-submitted economic models are scrutinized by the EGP before pERC makes its final funding recommendations. When compared with EGP-calculated estimates, manufacturers tend to overestimate the cost-effectiveness of their therapies when submitting economic models to pCODR. Although certain methodological issues are still common in manufacturer-submitted models, revision rates are high for most issues raised by the EGP. Given the ongoing methodological issues identified in this analysis and in previous studies, one natural option to consider is whether payers, such as pCODR, should directly construct economic models instead of modifying manufacturer submissions. However, this would require manufacturers to provide proprietary data access to pCODR, which is unlikely. Furthermore, pCODR would require greater resources to construct economic models for every drug submission. Because of resource limitations, it is currently feasible and is more convenient for pCODR to revise existing models from manufacturers rather than to build their own. Therefore, manufacturers should continue submitting economic models in accordance with the published guidelines. To improve the quality of these submitted models, and to better align EGP and manufacturer values, pCODR should continue revising their submission guidelines, highlighting and proposing solutions to commonly found methodological issues in submitted economic models. While manufactures tend to follow these pCODR guidelines, there is still room for interpretation, which can result in the model being revised by the EGP. For instance, the lack of consensus on what is considered a life-time horizon for specific cancers may currently result in model projections that do not reflect the clinical reality. This extrapolation of time horizon is a recurring issue identified in this analysis and in other literature. As manufacturers increasingly use surrogate endpoints and shorter follow-up times in clinical trials, potentially to gain early regulatory approval, correcting the time horizon issue remains an ongoing challenge. A potential solution to tackle this issue is implementing a process for lifecycle revaluation. For instance, when clinical trial data becomes mature, manufacturers should be encouraged to re-evaluate their initial model projections to see if they fit with the current data.

## Figures and Tables

**Table 1:** Key characteristics of the included submissions.

	Number (%) of Submissions
Year of pCODR Recommendation Issued	
2011	0 (0)
2012	3 (4)
2013	9 (12)
2014	7 (10)
2015	18 (25)
2016	10 (14)
2017	13 (18)
2018	13 (18)
Type of Therapy	
Chemotherapy	5 (7)
Targeted therapy	50 (68)
Immunotherapy	17 (23)
Others	1 (1)
Route of Administration	
Oral	34 (47)
Intravenous	39 (53)
Cancer Type	
Breast	5 (7)
Endocrine	2 (3)
Gastrointestinal	10 (14)
Genitourinary	4 (5)
Gynecology	3 (4)
Head and neck	1 (1)
Leukemia	12 (16)
Lung	10 (14)
Lymphoma	3 (4)
Myeloma	8 (11)
Sarcoma	3 (4)
Skin and melanoma	9 (12)
Other	3 (4)
pERC Final Recommendation	
Positive, without conditions	3 (4)
Positive, with conditions	59 (81)
Negative	11 (15)

pERC = pCODR Expert Review Committee.

## Supplementary Materials

The following are available online at https://www.mdpi.com/1718-7729/28/1/60/s1, Table S1: Characteristics of the included submission. Table S2: Economic values of the included submissions. Table S3: Summary Statistics for Manufacturer submitted and EGP-reanalyzed ICERs ($/QALY). Figure S1: Average ICERs generated by the manufacturer-submitted and EGP-re-analyzed economic models. Figure S2: Change in manufacturer-submitted and EGP-reanalysed ICERs over time. Cost is presented in CAD.
