# Supplementary material for: Comparing Manufacturer Submitted and Pan-Canadian Oncology Drug Review Reanalysed Incremental Cost-Effectiveness Ratios for Novel Oncology Drugs
Source: Curr Oncol. 2021 Jan 20;28(1):606–18. doi: 10.3390/curroncol28010060 (PMC7924399; doi:10.3390/curroncol28010060)

# Supplemental Materials for
## Comparing manufacturer submitted and pCODR reanalysed ICERs for novel oncology drugs

R. Saluja; T. Jiao, L. Koshy, M. Cheung MD, and K.K.W. Chan

## Listing of Supplemental Material(s):

Supplemental Table 1: Characteristics of the included submissions
Supplemental Table 2: Economic values of the included submissions
Supplemental Table 3: Summary Statistics for Manufacturer submitted and EGP-reanalyzed ICERs ($/QALY)
Supplemental Figure 1: Average ICERs generated by the manufacturer-submitted and EGP-re-analyzed economic models.
Supplemental Figure 2: Change in manufacturer-submitted and EGP-reanalysed ICERs over time. Cost is presented in CAD.

**Supplementary Table 1.** Characteristics of the included submissions

| Drug | pCODR # | Route | Indication | Date of pCODR Final Recommendation | pERC Recommendation |
|---|---|---|---|---|---|
| **2012** | | | | | |
| Sunitinib malate | 10004 | Oral | Pancreatic neuroendocrine tumours | 3-May-12 | Pos. conditional |
| Everolimus | 10007 | Oral | Pancreatic neuroendocrine tumours | 30-Aug-12 | Pos. conditional |
| Pazopanib Hydrochloride | 10009 | Oral | Soft Tissue Sarcoma (STS) | 29-Nov-12 | Negative |
| **2013** | | | | | |
| Ruxolitinib | 10012 | Oral | Myelofibrosis | 14-Jan-13 | Pos. conditional |
| Bortezomib | 10016 | IV | Multiple Myeloma | 25-Mar-13 | Pos. unconditional |
| Crizotinib | 10008 | Oral | Advanced Non-Small Cell Lung Cancer | 2-May-13 | Pos. Conditional |
| Pertuzumab | 10018 | IV | Metastatic Breast Cancer | 1-Aug-13 | Pos. Conditional |
| Abiraterone acetate | 10028 | Oral | Metastatic castration resistant prostate cancer | 22-Oct-13 | Pos. Conditional |
| Lenalidomide | 10029 | Oral | Multiple Myeloma | 22-Oct-13 | Pos. Conditional |
| Trametinib | 10030 | Oral | Metastatic Melanoma | 22-Oct-13 | Pos. Conditional |
| Pemetrexed | 10027 | IV | Advanced Non-Squamous Non Small Cell Lung Cancer | 19-Nov-13 | Pos. Conditional |
| Dabrafenib | 10025 | Oral | Metastatic Melanoma | 5-Dec-13 | Pos. Conditional |
| **2014** | | | | | |
| Cetuximab | 10031 | IV | Metastatic Colorectal Cancer | 10-Jan-14 | Negative |
| Arsenic Trioxide | 10033 | IV | Acute Promyelocytic Leukemia | 18-Feb-14 | Pos. Unconditional |
| Regorafenib | 10034 | Oral | Gastrointestinal Stromal Tumours | 2-May-14 | Pos. conditional |
| Afatinib | 10032 | Oral | Advanced Non Small Cell Lung Cancer | 2-May-14 | Pos. Conditional |
| Pomalidomide | 10036 | Oral | Multiple Myeloma | 31-Jul-14 | Pos. Conditional |
| Nab-paclitaxel | 10037 | IV | Metastatic Pancreatic Cancer | 23-Sep-14 | Pos. Conditional |
| Ipilimumab | 10042 | IV | First Line Advanced Melanoma | 22-Dec-14 | Pos. Conditional |
| **2015** | | | | | |
| Obinutuzumab | 10041 | IV | Chronic Lymphocytic Leukemia | 27-Jan-15 | Pos. Unconditional |
| Ofatumumab | 10038 | IV | Chronic Lymphocytic Leukemia | 29-Jan-15 | Negative |
| Ibrutinib | 10043 | Oral | Chronic Lymphocytic Leukemia/ Small Lymphocytic Lymphoma (previously treated) | 5-Mar-15 | Pos. Conditional |
| Bevacizumab | 10045 | IV | Cervical Cancer | 23-Mar-15 | Pos. Conditional |
| Bosutinib | 10039 | Oral | Chronic Myeloid Leukemia | 21-Apr-15 | Pos. Conditional |
| Romidepsin | 10048 | IV | Peripheral T-Cell Lymphoma | 19-May-15 | Pos. Conditional |
| Bevacizumab | 10047 | IV | Ovarian Cancer | 4-Jun-15 | Pos. Conditional |
| Enzalutamide | 10044 | Oral | First Line Metastatic Castration-Resistant Prostate Cancer | 22-Jun-15 | Pos. conditional |
| Siltuximab | 10052 | IV | Multicentric Castleman's Disease (MCD) | 22-Jun-15 | Pos. Conditional |
| Pertuzumab | 10050 | IV | Neoadjuvant Breast Cancer | 16-Jul-15 | Negative |
| Sorafenib | 10049 | Oral | Metastatic Progressive Differentiated Thyroid Carcinoma (DTC) | 16-Jul-15 | Negative |
| Bevacizumab | 10055 | IV | Metastatic Colorectal Cancer | 21-Jul-15 | Pos. Conditional |
| Crizotinib | 10054 | Oral | First Line ALK Positive Advanced NSCLC | 21-Jul-15 | Pos. conditional |
| Dabrafenib & Trametinib combo | 10053 | Oral | Metastatic Melanoma | 21-Jul-15 | Pos. Conditional |

| | | | | | |
|---|---|---|---|---|---|
| Ponatinib | 10056 | Oral | Chronic Myeloid Leukemia/ Acute Lymphoblastic Leukemia | 1-Oct-15 | Pos. Conditional |
| Ramucirumab | 10059 | IV | Metastatic Gastric Cancer or Gastro-Esophageal Junction Adenocarcinoma | 29-Oct-15 | Pos. Conditional |
| Pembrolizumab | 10058 | IV | Metastatic Melanoma | 16-Nov-15 | Pos. Conditional |
| Lenalidomide | 10061 | Oral | Multiple Myeloma (newly diagnosed) | 3-Dec-15 | Pos. Conditional |
| **2016** | | | | | |
| Ruxolitinib | 10065 | Oral | Polycythemia vera | 3-Mar-16 | Pos. Conditional |
| Nivolumab | 10063 | IV | Metastatic Melanoma | 1-Apr-16 | Pos. Conditional |
| Nivolumab | 10069 | IV | Non-Small Cell Lung Cancer | 3-Jun-16 | Pos. Conditional |
| Carfilzomib (with lenalidomide) | 10067 | IV | Multiple Myeloma | 21-Jun-16 | Pos. Conditional |
| Cobimetinib | 10070 | Oral | Metastatic Melanoma | 30-Jun-16 | Pos. Conditional |
| Trabectedin | 10071 | IV | Metastatic Liposarcoma or Leiomyosarcoma | 5-Aug-16 | Negative |
| Nivolumab | 10074 | IV | Metastatic Renal Cell Carcinoma | 1-Sep-16 | Pos. Conditional |
| Pembrolizumab | 10077 | IV | Non-Small Cell Lung Cancer (Second Line or Beyond) | 3-Nov-16 | Pos. conditional |
| Ibrutinib | 10085 | Oral | Chronic Lymphocytic Leukemia/Small Lymphocytic Lymphoma (previously untreated) | 3-Nov-16 | Pos. Conditional |
| Palbociclib (Resubmission) | 10093 | Oral | Advanced Breast Cancer | 21-Nov-16 | Pos. Conditional |
| **2017** | | | | | |
| Ceritinib (Resubmission) | 10094 | Oral | Non-Small Cell Lung Cancer | 21-Mar-17 | Pos. Conditional |
| Carfilzomib | 10084 | IV | Multiple Myeloma (relapsed) | 30-Mar-17 | Pos. conditional |
| Vandetanib | 10090 | Oral | Medullary Thyroid Cancer | 30-Mar-17 | Pos. Conditional |
| Alectinib | 10092 | Oral | Non-Small Cell Lung Cancer (with CNS metastases) | 4-May-17 | Negative |
| Ixazomib | 10088 | Oral | Multiple Myeloma | 29-Jun-17 | Negative |
| Pembrolizumab | 10101 | IV | Non-Small Cell Lung Carcinoma (First Line) | 23-Aug-17 | Pos. Conditional |
| Blinatumomab | 10099 | IV | Pediatric Acute Lymphoblastic Leukemia (ALL) | 23-Aug-17 | Pos. Conditional |
| Rituximab | 10102 | IV | Acute Lymphoblastic Leukemia | 31-Aug-17 | Negative |
| Nivolumab | 10095 | IV | Squamous Cell Carcinoma of the Head and Neck (SCCHN) | 31-Aug-17 | Pos. Conditional |
| Blinatumomab (Resubmission) | 10097 | IV | Adult Acute Lymphoblastic Leukemia (ALL) | 31-Aug-17 | Pos. Conditional |
| Olaparib (Resubmission) | 10103 | Oral | Ovarian Cancer | 20-Sep-17 | Pos. Conditional |
| Daratumumab | 10104 | IV | Multiple Myeloma (second-line or beyond) | 5-Oct-17 | Pos. Conditional |
| Nivolumab & Ipilimumab in combo | 10098 | IV | Metastatic Melanoma | 30-Nov-17 | Pos. Conditional |
| **2018** | | | | | |
| Irinotecan Liposome | 10107 | IV | Metastatic Pancreatic Cancer | 5-Jan-18 | Pos. Conditional |
| Fulvestrant | 10110 | IV | Locally Advanced or Metastatic Breast Cancer | 1-Feb-18 | Pos. Conditional |
| Brentuximab Vedotin | 10116 | IV | Hodgkin's Lymphoma at high risk of relapse or progression post-ASCT | 21-Feb-18 | Pos. Conditional |
| Venetoclax | 10105 | Oral | Chronic Lymphocytic Leukemia | 2-Mar-18 | Pos. Conditional |
| Pembrolizumab | 10117 | IV | Metastatic Urothelial Carcinoma | 2-Mar-18 | Pos. Conditional |
| Avelumab | 10124 | IV | Metastatic Merkel Cell Carcinoma | 21-Mar-18 | Pos. Conditional |
| Panitumumab | 10118 | IV | Left Sided Metastatic Colorectal Cancer | 29-Mar-18 | Negative |
| Alectinib | 10114 | Oral | Locally advanced or metastatic non-small cell lung cancer (second line) | 29-Mar-18 | Pos. Conditional |

| | | | | | |
|---|---|---|---|---|---|
| Regorafenib | 10119 | Oral | Unresectable Hepatocellular Carcinoma (HCC) | 18-Apr-18 | Pos. conditional |
| Olaratumab | 10111 | IV | Advanced Soft Tissue Sarcoma (STS) | 18-Apr-18 | Pos. conditional |
| Ribociclib | 10112 | Oral | Advanced or Metastatic Breast Cancer | 18-Apr-18 | Pos. conditional |
| Trifluridine and Tipiracil | 10122 | Oral | Metastatic Colorectal Cancer | 6-Jul-18 | Negative |
| Inotuzumab Ozogamicin | 10121 | IV | Acute Lymphoblastic Leukemia (ALL) | 6-Jul-18 | Pos. conditional |

Data organized based on year of pCODR final recommendation. pCODR, pan-Canadian Oncology Drug Review; Final Rec, final recommendation; pERC, pCODR Expert Review Committee; Pos. Conditional, positive conditional – pERC recommends funding conditional on certain metrics (i.e. cost-effectiveness) being improved to an acceptable level.

**Supplementary Table 2.** Economic values of the included submissions

| pCODR # | Drug | | Manufacturer submitted values | | | EGP reanalyzed values | | | | | |
|---|---|---|---|---|---|---|---|---|---|---|---|
| | **Experimental** | **Comparator** | **ICER ($/QALY)** | **ΔC ($)** | **ΔE (QALY)** | **LL ICER ($/QALY)** | **LL ΔC ($)** | **LL ΔE (QALY)** | **UL ICER ($/QALY)** | **UL ΔC ($)** | **UL ΔE (QALY)** |
| **2012** | | | | | | | | | | | |
| 10004 | Sunitinib malate | Placebo + BSC | 79,765 | 55,806 | 0.70 | 204,559 | 46,601 | 0.23 | 268,055 | 45,598 | 0.17 |
| 10007 | Everolimus | Placebo + BSC | 111,805 | 90,247 | 0.81 | 165,129 | 78,696 | 0.48 | 273,781 | 69,538 | 0.25 |
| 10009 | Pazopanib Hydrochloride | Placebo | 143,778 | 25,635 | 0.18 | 146,950 | 25,555 | 0.17 | 167,782 | 29,178 | 0.17 |
| **2013** | | | | | | | | | | | |
| 10012 | Ruxolitinib | BAT | 101,207 | 83,246 | 0.82 | 276,191 | 20,360 | 0.070 | 383,686 | 21,620 | 0.060 |
| 10016 | Bortezomib | SOC | 131,100 | 50,500 | 0.37 | 130,874 | 47,843 | 0.37 | 271,642 | 99,303 | 0.37 |
| N/A | Crizotinib | SOC | 124,472 | 180,973 | 1.45 | 124,472 | 180,973 | 1.45 | 246,117 | 118,764 | 0.48 |
| 10018 | Pertuzumab | Trastuzumab + Docetaxel | 238,014 | 120,287 | 0.51 | 262,263 | 117,932 | 0.45 | 303,726 | 100,699 | 0.33 |
| 10028 | Abiraterone acetate | Prednisone | 128,197 | 44,844 | 0.35 | 128,197 | 44,844 | 0.35 | 258,428 | 44,642 | 0.17 |
| 10029 | Lenalidomide | Placebo | 158,129 | 279,657 | 1.77 | 171,702 | 279,032 | 1.77 | 183,366 | 279,657 | 1.53 |
| 10030 | Trametinib | Vemurafenib | Dominant | -27,956 | 0.12 | 104,663 | 27,677 | 0.26 | 391,708 | 27,321 | 0.071 |
| 10027 | Pemetrexed | SOC | 143,261 | 36,396 | 0.25 | 170,272 | 37,460 | 0.21 | 173,864 | 38,250 | 0.22 |
| 10025 | Dabrafenib | Dacarbazine | 245,245 | 74,452 | 0.30 | 245,245 | 74,471 | 0.30 | 264,156 | 74,452 | 0.28 |
| 2014 | | | | | | | | | | | |
| 10031 | Cetuximab | Bevacizumab+ FOLFOX | 28,546 | 5,868 | 0.21 | 76,591 | 8,631 | 0.11 | 233,500 | 10,909 | 0.047 |
| 10033 | Arsenic Trioxide | All-trans retinoic + chemotherapy | 20,443 | 38,188 | 1.87 | 13,338 | 32,148 | 2.41 | 80,263 | 35,809 | 0.45 |
| 10034 | Regorafenib | BSC | 104,660 | 61,286 | 0.59 | 143,317 | 56,592 | 0.41 | 205,299 | 64,197 | 0.31 |
| 10032 | Afatinib | Gefitinib | 72,153 | 15,153 | 0.21 | 39,060 | 574 | 0.015 | 211,189 | 540 | 0.0026 |
| 10036 | Pomalidomide | BSC + HDex | 84,476 | 44,858 | 0.53 | 132,217 | 70,208 | 0.53 | 173,430 | 67,397 | 0.39 |
| 10037 | Nab-paclitaxel | Gemcitabine | 155,549 | 20,030 | 0.13 | 182,714 | 22,900 | 0.13 | 192,995 | 22,900 | 0.12 |
| 10042 | Ipilimumab | Dacarbazine | 151,014 | 111,419 | 0.74 | 165,389 | 108,880 | 0.53 | 197,382 | 110,343 | 0.67 |
| 2015 | | | | | | | | | | | |
| 10041 | Obinutuzumab | Chlorambucil | 30,844 | 30,065 | 0.98 | 32,369 | 29,958 | 0.93 | 49,823 | 29,240 | 0.59 |
| 10038 | Ofatumumab | Chlorambucil | 68,647 | 27,866 | 0.41 | 106,012 | 26,246 | 0.25 | 162,897 | 32,500 | 0.20 |
| 10043 | Ibrutinib | SOC | 124,954 | 148,364 | 1.19 | 80,941 | 185,089 | 1.94 | 382,134 | 117,601 | 0.31 |
| 10045 | Bevacizumab | Chemotherapy | 145,957 | 44,228 | 0.30 | 157,829 | 43,872 | 0.28 | 245,452 | 47,107 | 0.19 |
| 10039 | Bosutinib | Interferon | 45,955 | 101,954 | 2.22 | 44,198 | 60,013 | 0.42 | 146,587 | 62,047 | 1.56 |
| 10048 | Romidepsin | SOC | 186,253 | 102,266 | 0.55 | 217,588 | 105,224 | 0.48 | 387,056 | 144,434 | 0.32 |
| 10047 | Bevacizumab | Carboplatin + Paclitaxel | 96,261 | 36,021 | 0.37 | 87,033 | 36,943 | 0.42 | 113,473 | 35,158 | 0.32 |
| 10044 | Enzalutamide | BSC + docetaxel | 109,397 | 72,807 | 0.67 | 125,424 | 60,433 | 0.27 | 224,266 | 65,108 | 0.52 |
| 10052 | Siltuximab | Placebo + BSC | 204,332 | 294,782 | 1.44 | 232,663 | 294,782 | 1.27 | 648,163 | 195,047 | 0.30 |

| | | | | | | | | | | | |
|---|---|---|---|---|---|---|---|---|---|---|---|
| 10050 | Pertuzumab | Trastuzumab + Docetaxel | 25,388 | 7,879 | 0.31 | 17,103 | 6,585 | 0.39 | 27,550 | 8,550 | 0.31 |
| 10049 | Sorafenib | BSC | 142,843 | 74,527 | 0.52 | 189,647 | 79,609 | 0.42 | 206,945 | 80,148 | 0.38 |
| 10055 | Bevacizumab | Chemotherapy | 212,938 | 54,007 | 0.25 | 212,938 | 54,007 | 0.25 | 309,763 | 53,036 | 0.17 |
| 10054 | Crizotinib | SOC | 153,597 | 37,366 | 0.24 | 173,570 | 36,548 | 0.21 | 285,299 | 37,387 | 0.13 |
| 10053 | Dabrafenib & Trametinib combo | Vemurafenib | 332,129 | 114,493 | 0.35 | 323,454 | 46,052 | 0.14 | 446,238 | 153,830 | 0.37 |
| 10056 | Ponatinib | Hydroxyurea | 68,454 | 248,656 | 3.63 | 94,518 | 241,446 | 2.41 | 100,065 | 375,860 | 3.98 |
| 10059 | Ramucirumab | Placebo + Paclitaxel | 332,628 | 29,812 | 0.09 | 432,159 | 38,732 | 0.09 | 490,437 | 43,955 | 0.09 |
| 10058 | Pembrolizumab | Ipilimumab | 52,829 | 39,099 | 0.74 | 114,389 | 63,060 | 0.55 | 151,369 | 56,320 | 0.37 |
| 10061 | Lenalidomide | Bortezomib | 100,784 | 102,826 | 1.02 | Dominated | 146,793 | 0 | Dominated | 150,304 | 0 |
| **2016** | | | | | | | | | | | |
| 10065 | Ruxolitinib | BAT | 156,250 | 211,240 | 1.35 | 282,785 | 169,575 | 0.60 | 284,555 | 170,274 | 0.60 |
| 10063 | Nivolumab | Ipilimumab | 94,176 | 80,234 | 0.85 | 120,851 | 104,067 | 0.89 | 198,776 | 145,608 | 0.52 |
| 10069 | Nivolumab | Docetaxel | 133,520 | 84,918 | 0.64 | 183,386 | 80,014 | 0.44 | 236,851 | 80,014 | 0.34 |
| 10067 | Carfilzomib (with lenalidomide) | Lenalidomide + Dexamethasone | 201,216 | 152,034 | 0.76 | 270,652 | 155,134 | 0.57 | 347,640 | 174,431 | 0.50 |
| 10070 | Cobimetinib | Vemurafenib | 317,648 | 157,117 | 0.50 | 314,268 | 157,117 | 0.50 | 426,815 | 156,853 | 0.37 |
| 10071 | Trabectedin | Dacarbazine | 167,863 | 25,019 | 0.15 | 318,519 | 28,626 | 0.090 | 583,041 | 52,458 | 0.09 |
| 10074 | Nivolumab | Everolimus | 131,349 | 63,185 | 0.48 | 186,312 | 89,625 | 0.48 | 242,521 | 89,625 | 0.37 |
| 10077 | Pembrolizumab | Docetaxel | 143,730 | 76,742 | 0.53 | 149,342 | 71,649 | 0.48 | 254,945 | 68,441 | 0.27 |
| 10085 | Ibrutinib | Chlorambucil | 101,405 | 291,214 | 2.87 | 141,616 | 249,509 | 1.76 | 233,945 | 178,941 | 0.77 |
| 10093 | Palbociclib (Resubmission) | Letrozole | 310,007 | 198,623 | 0.64 | 295,925 | 174,484 | 0.65 | 745,785 | 227,517 | 0.26 |
| **2017** | | | | | | | | | | | |
| 10094 | Ceritinib (Resubmission) | Pemtrexed or Docetaxel or BSC or historical controls | 118,676 | 70,293 | 0.59 | 159,750 | 75,766 | 0.47 | 208,377 | 98,829 | 0.47 |
| 10084 | Carfilzomib | Bortezomib + Dexamethasone | 192,997 | 147,701 | 0.77 | 261,648 | 157,554 | 0.60 | 294,931 | 163,029 | 0.55 |
| 10090 | Vandetanib | BSC | 285,627 | 128,566 | 0.45 | 314,801 | 131,250 | 0.42 | 434,852 | 128,963 | 0.30 |
| 10092 | Alectinib | SOC | 108,958 | 156,501 | 1.44 | 67,993 | 127,124 | 1.96 | 417,128 | 185,878 | 0.42 |
| 10088 | Ixazomib | Lenalidomide + Dexamethasone | 378,299 | 350,680 | 0.93 | 464,746 | 348,272 | 0.75 | 1,751,236 | 345,495 | 0.20 |
| 10101 | Pembrolizumab | SOC | 99,392 | 98,298 | 0.99 | 111,769 | 107,632 | 0.96 | 154,273 | 103,406 | 0.67 |
| 10099 | Blinatumomab | Chemotherapy | 15,940 | 67,913 | 4.26 | 6,577 | 48,572 | 7.38 | 100,948 | 112,363 | 1.11 |
| 10102 | Rituximab | Chemotherapy | 39,181 | 45,259 | 1.16 | 46,894 | 32,299 | 0.69 | 4,193,972 | 44,372 | 0.01 |
| 10095 | Nivolumab | Docetaxel | 67,616 | 30,816 | 0.46 | 109,743 | 37,682 | 0.34 | 145,855 | 33,802 | 0.23 |
| 10097 | Blinatumomab (Resubmission) | Chemotherapy | 72,488 | 158,183 | 2.18 | 223,060 | 158,224 | 0.71 | 971,327 | 158,270 | 0.16 |
| 10103 | Olaparib (Resubmission) | Watchful waiting | 243,249 | 258,015 | 1.06 | 195,112 | 251,171 | 1.29 | 421,637 | 257,020 | 0.61 |

| | | | | | | | | | | | |
|---|---|---|---|---|---|---|---|---|---|---|---|
| 10104 | Daratumumab | Lenalidomide + Dexamethasone | 181,212 | 539,113 | 2.97 | 165,496 | 622,746 | 3.76 | 594,144 | 422,874 | 0.71 |
| 10098 | Nivolumab & Ipilimumab in combo | Ipilimumab | 66,750 | 149,556 | 2.24 | 86,758 | 125,019 | 1.44 | 116,541 | 145,958 | 1.25 |
| **2018** | | | | | | | | | | | |
| 10107 | Irinotecan Liposome | 5-FU/LV | 182,719 | 23,871 | 0.13 | 326,774 | 43,039 | 0.15 | 335,528 | 44,496 | 0.13 |
| 10110 | Fulvestrant | Anastrozole | 32,361 | 41,405 | 1.28 | 33,476 | 52,416 | 1.24 | 185,631 | 35,095 | 0.19 |
| 10116 | Brentuximab Vedotin | SOC | 26,303 | 113,900 | 4.33 | 105,383 | 125,510 | 1.19 | 139,286 | 123,999 | 0.89 |
| 10105 | Venetoclax | SOC | 124,050 | 184,319 | 1.49 | 139,074 | 359,461 | 2.59 | 1,474,649 | 69,893 | 0.047 |
| 10117 | Pembrolizumab | Paclitaxel/Docetaxel | 126,150 | 94,317 | 0.75 | 217,954 | 89,225 | 0.41 | 285,514 | 84,631 | 0.30 |
| 10124 | Avelumab | Chemotherapy | 57,051 | 111,214 | 1.95 | 84,155 | 97,282 | 1.16 | 97,962 | 126,533 | 1.13 |
| 10118 | Panitumumab | Bevacizumab + FOLFOX | 110,414 | 36,118 | 0.33 | 126,389 | 40,686 | 0.32 | 275,255 | 18,594 | 0.068 |
| 10114 | Alectinib | Chemotherapy | 84,444 | 123,767 | 1.47 | 87,357 | 120,560 | 1.38 | 159,544 | 152,170 | 0.95 |
| 10119 | Regorafenib | BSC | 138,322 | 34,194 | 0.25 | 152,657 | 34,084 | 0.25 | 175,700 | 40,411 | 0.23 |
| 10111 | Olaratumab | Doxorubicin | 175,001 | 127,075 | 0.73 | 224,817 | 128,682 | 0.57 | 263,340 | 124,109 | 0.47 |
| 10112 | Ribociclib | Letrozole | 136,140 | 231,283 | 1.70 | 175,827 | 231,283 | 1.32 | 204,805 | 164,261 | 0.84 |
| 10122 | Trifluridine and Tipiracil | BSC | 96,971 | 16,688 | 0.17 | 123,849 | 18,141 | 0.15 | 130,314 | 19,088 | 0.15 |
| 10121 | Inotuzumab Ozogamicin | Chemotherapy | 91,841 | 120,883 | 1.32 | 178,801 | 109,370 | 0.75 | 335,752 | 140,560 | 0.67 |

Data organized based on year of pCODR final recommendation. All costs are in Canadian dollars. pCODR, pan-Canadian Oncology Drug Review; ICER, incremental cost-effectiveness ratio; ΔC, incremental cost; ΔE, incremental effectiveness; LL, lower limit; UL, upper limit; QALY, quality-adjusted life year, BSC, best supportive care; SOC, Standard of care; BAT, Best available therapy; EGP, Economic guidance panel.

**Supplementary Table 3.** Summary Statistics for Manufacturer submitted and EGP-reanalyzed **ICERs ($/QALY)**

| | **Mean** | **Std Dev.** | **Median** | **Min** | **Max** |
|---|---|---|---|---|---|
| **Manufacturer Submitted** | 134,241 | 79,803.45 | 124,713 | 15,940 | 378,299 |
| **EGP Lower Limit Estimates** | 166,382 | 93,545.76 | 155,243 | 65,77 | 464,746 |
| **EGP Upper Limit Estimates** | 365,018 | 533,382 | 245,784.50 | 27,550 | 4,193,972 |

ICER, Incremental Cost-Effectiveness Ratio; EGP, Economic Guidance Panel.

**Supplementary Figure 1.** Average ICERs generated by the manufacturer-submitted and EGP-re-analyzed economic models.

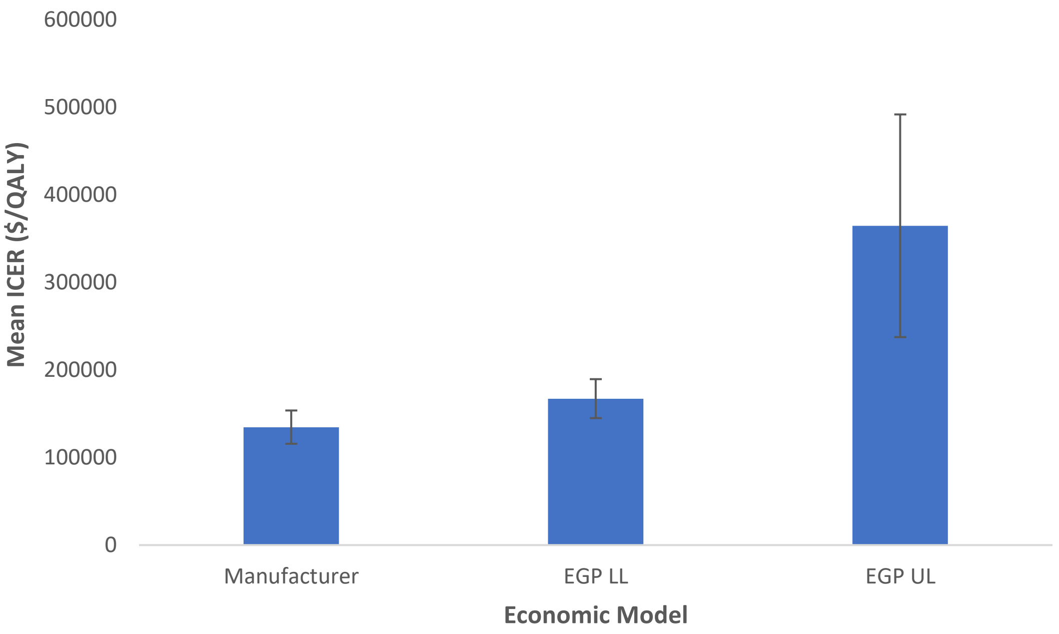


Note: Excluded ICERs for all three economic models if 1 economic model deemed the drug as dominant (1) or dominated (1) and did not provide a numerical ICER value. Whiskers represent 95% confidence intervals. ICER, incremental cost-effectiveness ratio; EGP, Economic Guidance Panel; LL, lower limit; UL, upper limit

**Supplementary Figure 2.** Change in manufacturer-submitted and EGP-reanalysed ICERs over time. Cost is presented in CAD.

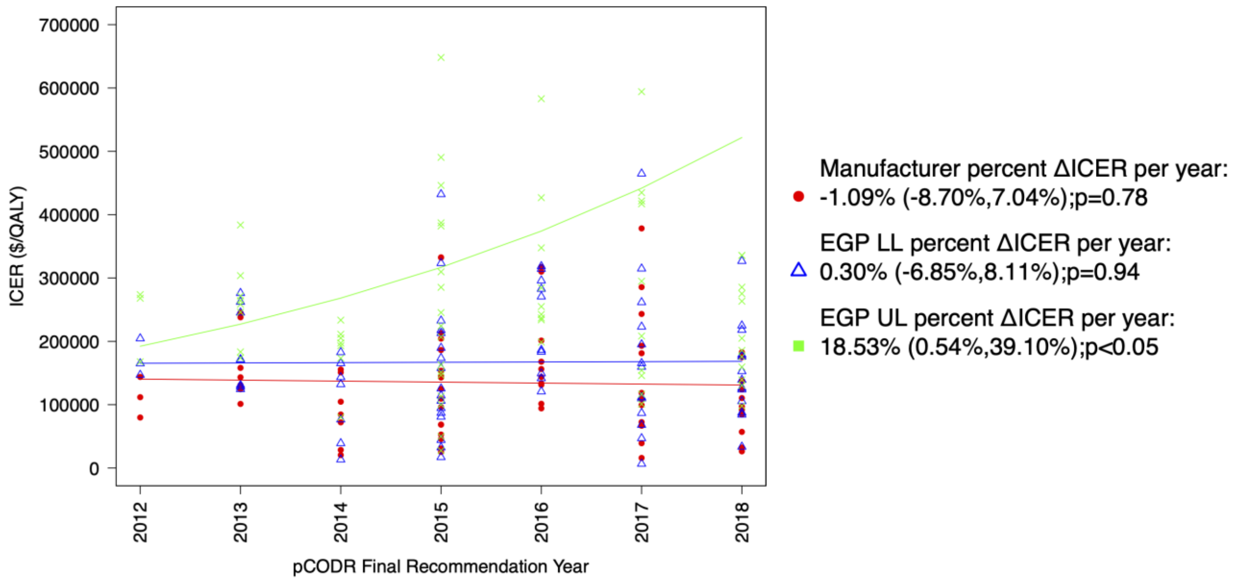

Supplement: Supplementary file 1 [file curroncol-28-00060-s001.pdf]
